# Efficiency of quality of triggering system and pressurization of home care ventilators. a bench

**DOI:** 10.1186/2197-425X-3-S1-A272

**Published:** 2015-10-01

**Authors:** L Baboi, S Guegan, C Guérin

**Affiliations:** Hôpital de la Croix Rousse, Lyon, France; Ecole des Haute Etudes Ingenieur, Lille, France; Hopital de la Croix Rousse, Lyon, France; INSERM UMR 955, Créteil, France

## Introduction

Turbine home care ventilators can be used in the hospital to manage patients with hypercapnic acute respiratory failure. The goal of this study was to assess efficiency of quality of triggering system and pressurization of these ventilators.

## Methods

Astral 150, Elisée 150, Trilogy 200, Monnal T50 (double limb circuit) and Evita XL were set in pressure support (PS) 15 cm H2O with positive end expiratory pressure (PEEP) 5 cmH2O. In each ventilator the specific leak compensation system was activated. Each ventilator was used with its optimal inspiratory triggering system facing leaks. Inspiratory trigger was set at maximum sensitivity avoiding autotriggering. The ventilators were connected to ASL 5000 lung model set in a condition mimicking COPD patient (compliance 75 ml/cmH2O, inspiratory and expiratory airways resistance 15 and 25 cmH2O/L/s, respectively). We compared low, moderate and strong inspiratory efforts (-3, -6 and -12 cm H2O muscular pressure, respectively) with and without calibrated non intentional leak (NIL 20 L/min at 15 cm H2O). Pressure time product (PTP) of the triggering system (PTPtrig) was measured over 10 breaths as the area subtended by the pressure over the time spent between onset of inspiratory effort and start of pressurization (time unsupported). PTP500 was measured between onset of inspiratory effort and 500 ms after. When time unsupported was greater than 500 ms (delayed effort) PTP500 was set to 0. Measured PTP500 was compared to ideal PTP500, which is equal to PS level achieved at the end of inspiration x 500 ms (PTP%ideal).

Values were expressed as mean ± SD. Comparisons were made by using one-factor ANOVA and multiple comparisons between ventilators by using Tukey test. Significant statistical threshold was set to P < 0.001 to take into account the number of statistical tests performed.

## Results

For PTPtrig with NIL, there were marked differences across ventilators for low and moderate efforts that were no longer present for strong effort (Figure). Without NIL, the results were in the same direction. Significant differences were observed between ventilators for PTP500 at each effort with or without NIL. The same was true for PTP500%ideal. As an example, for strong effort without NIL, PTP500%ideal averaged 70 ± 1% for Astral, 70 ± 2% for Elisée, 55 ± 1% for Evita XL, 50 ± 1% for Monnal T50 and 34 ± 4% for Trilogy (P < 0.001 between ventilators). With NIL, these values were 73 ± 12, 32 ± 40, 66 ± 29, 58 ± 26 and 35 ± 21 (P < 0.001 between ventilators), respectively.

**Figure 1:**
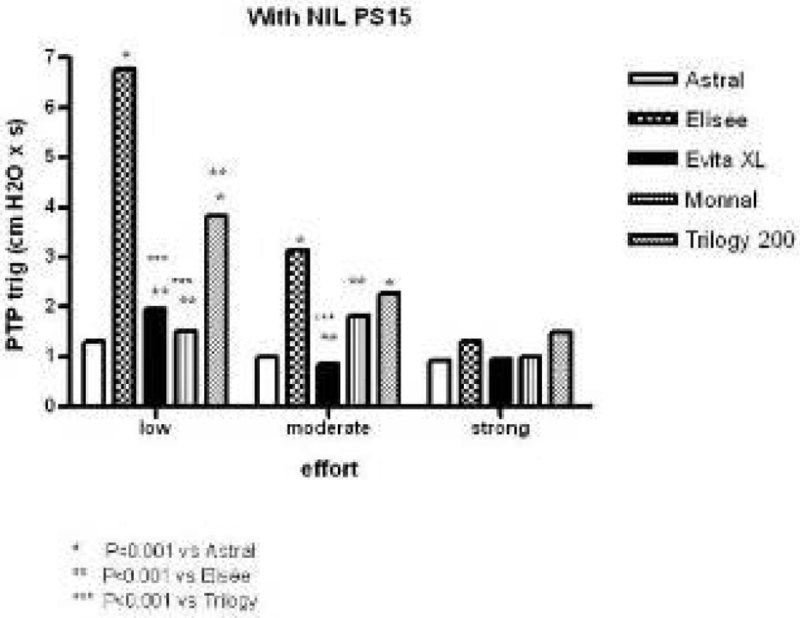
Figure 1

## Conclusions

There were marked differences in PTPtrig between ventilators that were also dependent on intensity of effort. The overall quality of PS mode as assessed in present study, was significantly different across the ventilators tested, with the Astral ventilator exhibiting best performance.

